# Guiametabolica.org: empowerment through internet tools in inherited metabolic diseases

**DOI:** 10.1186/1750-1172-7-53

**Published:** 2012-08-21

**Authors:** Manuel Armayones, M Antònia Vilaseca, Júlia Cutillas, Jordi Fàbrega, Jorge Juan Fernández, Mei García, Natàlia Egea, Modesta Pousada, Beni Gómez-Zuñiga, Jaume Pérez-Payarols, Rafael Artuch, Francesc Palau, Mercedes Serrano

**Affiliations:** 1 Psychology and Educational Sciences Deparment. Internet Interdisciplinary Institute (IN3) Psinet Research Group, Universitat Oberta de Catalunya, Barcelona, Spain; 2 Neurometabolic Unit, Hospital Sant Joan de Déu, Universidad de Barcelona, U-703 Barcelona, Spain and Centre for Biomedical Network Research on Rare Diseases (CIBERER), Instituto de Salud Carlos III, Barcelona, Spain; 3 Innovation, Research and Communication Departments, Hospital Sant Joan de Déu, Barcelona, Spain; 4 Associació catalana de fenilcetonúria i altres trastorns del metabolisme, Barcelona, Spain; 5 Instituto de Biomedicina de Valencia, CSIC, and Centre for Biomedical Network Research on Rare Diseases (CIBERER), Instituto de Salud Carlos III, Valencia, Spain

**Keywords:** Dietary treatment, E-patient, Inborn errors of metabolism, Inherited metabolic diseases, Patient empowerment

## Abstract

Web-based interventions are effective on the patient empowerment. *Guiametabolica.org* constitutes an interface for people involved in inherited metabolic diseases, trying to facilitate access to information and contact with professionals and other patients, offering a platform to develop support groups. *Guiametabolica.org* is widely considered for Spanish-speaking patients and caregivers with inherited metabolic diseases. Preliminary evaluations show changes in their habits, decrease in their senses of isolation and improvement regarding self-efficacy. Specific inherited metabolic diseases websites, especially participative websites, should be considered as a complement to more traditional clinical approaches. Their contribution lies in patient’s general well-being, without interfering with traditional care.

## Findings

Inherited metabolic diseases (IMD) are a very heterogeneous group of more than 500 rare diseases that mainly appear during childhood. The effectiveness of Web-based interventions on the patient empowerment, which is essential for patients with rare diseases for various reasons, has recently been proven [1-3]. *Guiametabolica.org* is a frequently-updated website in Spanish for people involved in IMD that facilitates access to information and contact with professionals and other similar patients, and offers a platform for developing support groups.

**Figure 1:**
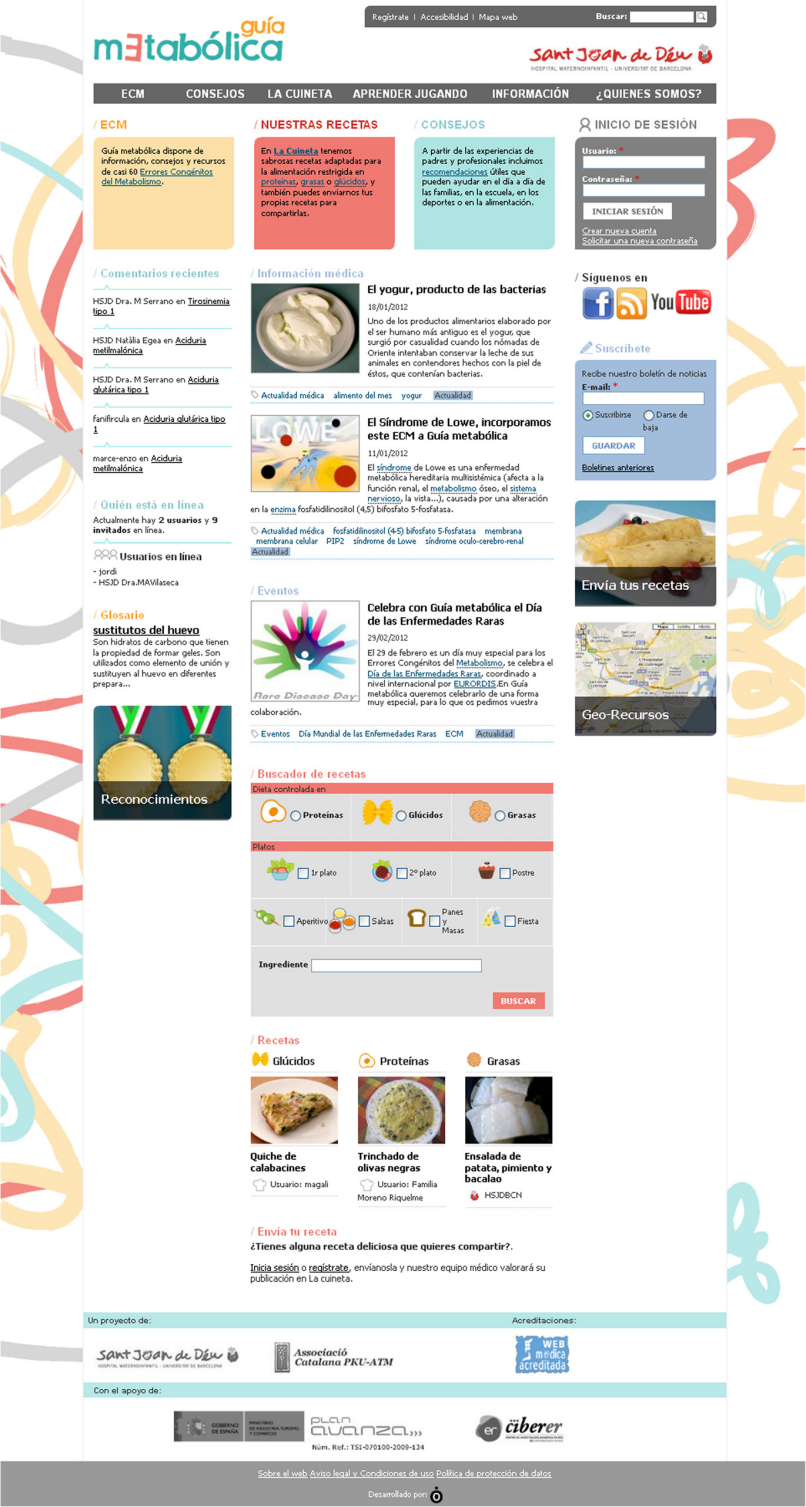
**Home page of www.guiametabolica.org**.

*Guiametabolica.org* offers (Figure 1): scientific information in easy jargon about clinical features, biochemical traits, genetics, treatment and prognosis of 56 IMD, translated abstracts of more than 300 articles, more than 100 tips and recommendations for daily life, 32 geolocated-resources, 172 specific recipes for controlled diets in proteins, carbohydrates and fat, and 6 stories for children in which IMD patients are the leading characters.

Through the comment field, *Guiametabolica.org* offers a chance to pose medical/nutritional questions to our clinical team, and to share experiences among families. The target population is estimated at about 62,000 people around the world, considering Spanish speaking people involved in IMD.

Aiming to evaluate our platform and to analyse the effect of the social network initiative in a public hospital setting and focalized in different rare diseases, we elaborated a questionnaire. E-patients and e-caregivers’ feelings and self-perceptions, as well as their developed skills and abilities, were analyzed as a measure of their empowerment process.

### *Guiametabolica.org* in the internet

**Figure 2:**
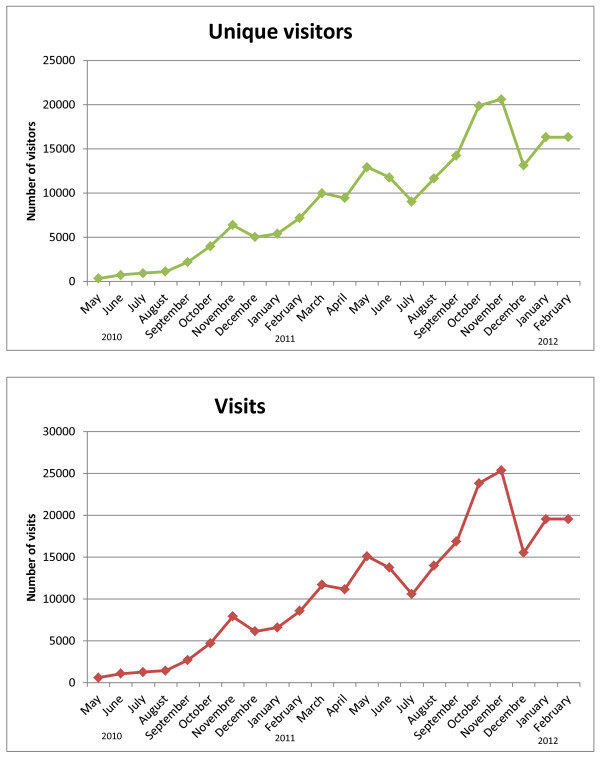
**Map view of Guiametabolica.org and top ten countries regarding visits to the website (from Google Analytics, February 20th, 2012)**.

**Figure 3:**
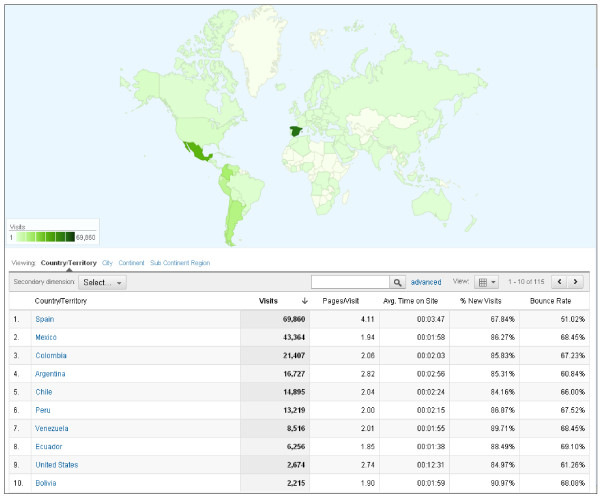
**Evolution of unique visitors and visits of Guiametabolica.org (from Google Analytics, February 20th, 2012)**.

In 22 months we registered 243,305 visits and 663,497 page views, from more than 100 countries, and with average time on the site of 2:45 minutes (Figure 2). Near 70% of the visitors came from Latin America (Figure 3). *Guiametabolica.org* registered 886 comments that have been handled by the nutritional/medical staff (Table 1). There is equilibrium between those comments regarding educational issues and those related to *Guiametabolica.org* function as a support group (Table 2).

**Table 1:** Typology of contributions

**Number**	***Kind of contribution***
	**Comments/Questions/Answers**
257	Medical/nutritional questions
324	Medical/nutritional answers from nutritional/medical staff
73	Medical/nutritional answers from other users
69	Requests to contact other patients/caregivers
163	Other kinds of intervention
	**Recipes**
133	Guiametabolica staff
40	Users/ Fundación Alicia/ Consumer Eroski
	**Geolocated Resources**
22	Guiametabolica staff
10	Users

**Table 2:** Analysis of users’ questions (20th December 2011)

**Comments/questions’ topics**	**Percentage**	**Comments**
Comments related to *Guiametabolica.org *function as a support group		
Requests to contact other patients/caregivers	17.5%	Frequent situation: a family with a young child suffering an IMD with plenty of uncertainties about future, demanding contact with older patients or parents
Patients’ experiences and reflections, tips or recommendations.	13.0%	All of them with a positive point of view
Questions about practical issues of the daily life	5.8%	They include help for travelling, shopping, offers of special foods not needed anymore, information about government aids for chronic patients…
Messages exclusively to thank and express feelings	14.8%	Messages of gratitude and feelings are also incorporated in the vast majority of comments
Comments regarding educational issues		
Information about their IMD	22.1%	
	Doubts about diagnostic process 5.8%	
	Doubts about treatments 5.4%	
	Biochemical and pathophysiological doubts 4.0%	
	Questions about clinical manifestations and evolution of disease 3.3%	
	Therapeutic options under investigation 2.2%	
	Questions about clinical/biochemical follow-up 1.4%	
Doubts about other transitory or concomitant situations and their treatment	10.2%	
Doubts about dietary treatments and specific recipes.	16.6%	

Helpfulness of the different categories of contents is represented in Table 3. Concerning educational issues, 93.6% of questionnaire responders discovered new information or increased their knowledge. Regarding changes in daily life, 50.1% changed or developed at least one new habit. Regarding feelings of solitude, 75.4% of the visitors acknowledged that they felt less lonely and 72.4% realized that they were doing well when getting in touch with others in the same situation.

**Table 3:** Users’ evaluation: Usefulness of contents

**Content**	Mean* (Standard Deviation)	**No response**
Specific recipes	3.42 (0.8)	14/81
Scientific/medical information	3.37 (0.9)	5/81
Medical/nutritional responses to comments	3.34 (1.1)	8/81
Tips and recommendations	3.28 (0.9)	6/81
News	3.16 (0.9)	10/81
Comments from other users	3.07 (1.0)	11/81
Tales and games	3.01 (1.0)	10/81
Geolocated resources	2.78 (1.1)	26/81

*Helpfulness of the different categories of contents was scored in a 1-to-4 Guttman scale (1 = strongly disagree, 4 = strongly agree) by the users.

## Conclusions

To the best of our knowledge, this is the first interactive website focused on IMD in Spanish providing plain language information about a large number of IMDs and offering online consultations with medical/nutritional staff.

There is probably an excess of health information on the Internet [4], especially true for prevalent diseases. However, regarding rare diseases there is a lack of information, particularly easy-to-understand information. *Guiametabolica.org* offers relevant and up-to-date information in easy jargon that is, in fact, considered one of the most useful contents by our visitors. A sense of personal empowerment is often achieved by acquiring relevant information and knowledge, especially when information is acquired through direct access to relevant sources [5,6]. Moreover, successful communication may be especially important for patients with chronic clinical conditions, such as IMD patients [7].

We believe that *Guiametabolica.org* role in some of the developing countries of Latin America, has to do with the limited resources that they have for health, particularly for rare diseases. We think that *Guiametabolica.org* can contribute to overcome the “health digital divide” providing users and professionals of developing countries useful information and tools to manage IMD diseases.

Users of *Guiametabolica.org* are very participative, probably due to the particular profile of our users; rather than e-patients, they are mainly e-caregivers (more than 80%). They are normally parents with a young child suffering an IMD, and then they are 30-to-50 years old, increasing their likelihood of being digital natives [8].

Furthermore, parents acting as e-caregivers could be more active and participative for two powerful reasons. The first one is related to strong filial affection, and the second one is due to the disconcerting situation of a child suffering a chronic illness. Caring for a child with chronic situation can become burdensome and can impact upon the physical and psychological health and, ultimately, the well-being of the caregivers [9]. In the case of rare diseases, this situation may lead to uneasiness and usually generates numerous questions that need responses, making the e-caregiver more participative, involved and committed [10].

Our preliminary data show a decrease in feelings of loneliness among our users, as well as a reduced level of self-criticism; taken together, those results probably mean that the web is having an impact in the psychological and emotional health of the users.

We believe that specific IMD websites, especially online support groups, should be considered as a complement to more traditional clinical approaches. *Guiametabolica.org* contribution lies in the effect that it has on people’s general well-being while not interfering with traditional care.

## Competing interests

The authors declare no financial or non-financial competing interest.

## Authors’ contribution

MA, MAV, JJF and MS contributed to the conception, design, organization and execution of the research project, and to the drafting of the manuscript. JC, MG, NG, MP and BGZ contributed to the acquisition of data, analysis and interpretation, and to the review and critique of the manuscript. FP, JPP and RA contributed to the review and critique of the manuscript. All authors reviewed and accepted the final version of the manuscript. The corresponding author is responsible for ensuring that author contributions and full disclosures appear on the submitted, revised, and final accepted manuscript.
